# Efficacy and safety of acupuncture combined Chinese herbal medicine for diabetic peripheral neuropathy: A protocol for systematic review and meta-analysis

**DOI:** 10.1097/MD.0000000000028086

**Published:** 2021-12-17

**Authors:** Yong Shi, Le Liu, Xuefeng Sun, Jundong Jiao

**Affiliations:** a School of Special Education, Changchun University, 6543 Weixing Road, Changchun, China; b Traditional Chinese Medicine College of Changchun University of Chinese Medicine, Changchun, China; c Nursing College of Changchun University of Chinese Medicine, Changchun, China; d Affiliated Hospital of Changchun University of Traditional Chinese Medicine, Changchun, China.

**Keywords:** acupuncture combined with Chinese herbal medicine, diabetic peripheral neuropathy, effectiveness, protocol, safety, systematic review

## Abstract

**Background:**

Acupuncture combined with Chinese herbal medicine has been widely utilized for pain management in patients with diabetic peripheral neuropathy (DPN). However, its results are still inconsistent, and no systematic review has specifically addressed this issue. Thus, this systematic review will comprehensively and systematically investigate the effectiveness and safety of acupuncture combined with Chinese herbal medicine for pain relief in DPN.

**Methods:**

Randomized controlled trials on acupuncture combined with Chinese herbal medicine treatment of DPN published before September of 2021 will be searched in 9 databases including Medline, Web of Science, PubMed, Cochrane Library, Excerpta Medica Database, Sinomed, China National Knowledge Infrastructure, WanFang, and China Science and Technology Journal Database. The methodological assessment performed using the risk of bias assessment tool of Cochrane, and the level of evidence quality for the main results will be evaluated by a recommended grading, evaluation, formulation, and evaluation system approach. Bayesian network meta-analysis will be conducted using STATA V.14.0 and WinBUGS V.1.4.3.

**Results:**

This study will provide a high-quality comprehensive evaluation of the safety of acupuncture combined with Chinese herbal medicine for treating DPN.

**Conclusion:**

This systematic review will evaluate the efficacy and safety of Chinese herbal medicine combined with acupuncture in the treatment of DPN, and provide the latest evidence for clinical application.

**Ethics and dissemination:**

The protocol of the systematic review does not require ethical approval because it does not involve humans. This article will be published in peer-reviewed journals and presented at relevant conferences.

Registration number: INPLASY2021100004.

## 1 Introduction

Diabetic peripheral neuropathy (DPN) is one of the diabetic microvascular complications. It is also one of the main causes of death in diabetes. The main clinical symptoms are limb pain, numbness, burning, or other abnormal feelings.^[1,2]^ In the late stage of DPN, muscle atrophy, weakness, foot ulceration, infection, gangrene, and even amputation. Research shows that when the duration of diabetes exceeds 15 years, 94% of patients will have varying degrees of neuropathy, and 25% of diabetic neuropathy patients can have serious consequences such as foot ulcers and amputation.^[3]^ The disease has brought a heavy burden to the patients and their families, and the patients themselves are even more miserable. At the same time, it has caused a huge economic burden to the state, society, and families.^[4]^

It is reported that the current treatment for diabetic peripheral neuropathy mainly consists of general treatment, symptomatic supportive treatment, and drug therapy, with unsatisfactory therapeutic effects. Acupuncture combined with Chinese herbal medicine has a good curative effect in delaying patients’ symptoms and improving the cure rate of the disease.^[5–7]^ Therefore, the rational use of acupuncture combined therapy in the early stage of diabetic peripheral neuropathy has important clinical significance for the rehabilitation of patients.^[8–10]^ In recent years, there has been a lot of literature about the combination of acupuncture and medicine for diabetic peripheral neuropathy in various databases.^[11–13]^ However, there is a lack of high-quality systematic reviews and meta-analysis. Therefore, this study will objectively compare and analyze the efficacy and safety of acupuncture combined with Chinese herbal medicine for diabetic peripheral neuropathy, which will provide a new basis for clinical treatment of diabetic peripheral neuropathy.

## 2 Study registration

This study has been registered on INPLASY2020100004. It has been reported according to the guideline of Preferred Reporting Items for Systematic Reviews and Meta-Analysis Protocol statement.

## 3 Methods and analysis

### 3.1 Inclusion criteria

#### 3.1.1 Type of studies

We will select clinical randomized controlled trials (RCTs) published before September of 2021, and without any regional and language restrictions. Animal studies, case reports, retrospective studies, and reviews will be excluded. About duplicate articles, we will prefer the 1 with more comprehensive data.^[14]^

#### 3.1.2 Type of participants

All participants were diagnosed with diabetic peripheral neuropathy, and gender, race, and age were not restricted.^[15]^

#### 3.1.3 Type of interventions

The intervention method of the treatment group was acupuncture combined with Chinese herbal medicine. There are no requirements for medication time, medication frequency, and drug dosage form. The control group received simple Western medicine treatment or a placebo.

#### 3.1.4 Type of outcomes

The main outcome measures included fasting blood glucose, postprandial blood glucose, and glycosylated hemoglobin.

The secondary outcomes included:

(1) Toronto clinical scoring system will be used for evaluation, including symptom score, sensory function score, and reflex score.
(2) Electromyography detection: the motor conduction velocity and terminal latency of the tibial nerve, common peroneal nerve, and median nerve were measured before and after treatment.
(3) Traditional Chinese Medicine syndrome score was established according to clinical symptoms and signs, and measured once before and after treatment.

### 3.2 Exclusion criteria

(1) Non-RCTs.
(2) None of the valid outcome indicators.
(3) Duplicated data.
(4) Invalid outcome indexes.

### 3.3 Data selection and extraction

#### 3.3.1 Electronic searches

The keywords such as “Chinese herbal medicine,” “Acupuncture,” “Diabetic neuropathy,” “Neuropathy,” “Peripheral neuropathy,” “Diabetic,” “Diabetic polyneuropathy,” “Diabetes mellitus,” and “Diabetic neuropathies” were used to search in the following electronic databases: Medline, Web of Science, PubMed, Cochrane Library, Excerpta Medica Database, Sinomed, China National Knowledge Infrastructure, WanFang, and China Science and Technology Journal Database, and there are no regional and language restrictions. We will consider the article published between database initiation and September 2021. The search strategy for PubMed is presented in the following Table 1.

**Table 1:** Search strategy for the PubMed database.

Number	Search terms
#1	diabetic neuropathy(all field)
#2	peripheral neuropathy(all field)
#3	diabetic polyneuropathy(all field)
#4	diabetic neuropathies(all field)
#5	neuropathy(all field)
#6	diabetic(all field)
#7	diabetes mellitus(all field)
#8	#1OR#2-7
#9	acupuncture(all field)
#10	acupuncture therapy(all field)
#11	scalp acupuncture(all field)
#12	fire needling(allfield)
#13	Intradermal needling(all field)
#14	ear acupuncture(all field)
#15	acupoint(all field)
#16	auricuar acupuncture(all field)
#17	electroacupuncture(all field)
#18	catgut embedding(all field)
#19	#9 OR #10-18
#20	Chinese medicine(all field)
#21	Traditional Chinese medicine(all field)
#22	Proprietary Chinese medicine(all field)
#23	Chinese herb medicine(all field)
#24	Proprietary Chinese medicine(all field)
#25	Chinese herbs(all field)
#26	#20 OR #21-26
#27	randomized controlled trial(all field)
#28	randomly(all field)
#29	controlled clinical trial(all field)
#30	randomized(all field)
#31	random allocation(all field)
#32	placebo(all field)
#33	single-blindmethod(all field)
#34	double-blindmethod(all field)
#35	trials(all field)
#36	Comparators
#37	allocation
#38	#27 OR #28-37
#39	#8 And #19 And #26 And #38

#### 3.3.2 Searching other resources

A review or meta-analysis of relevant RCT systems will be conducted via electronic search. We will also manually search the references of relevant articles that are not included in the electronic database to further identify eligible studies.

### 3.4 Selection of studies

**Figure 1:**
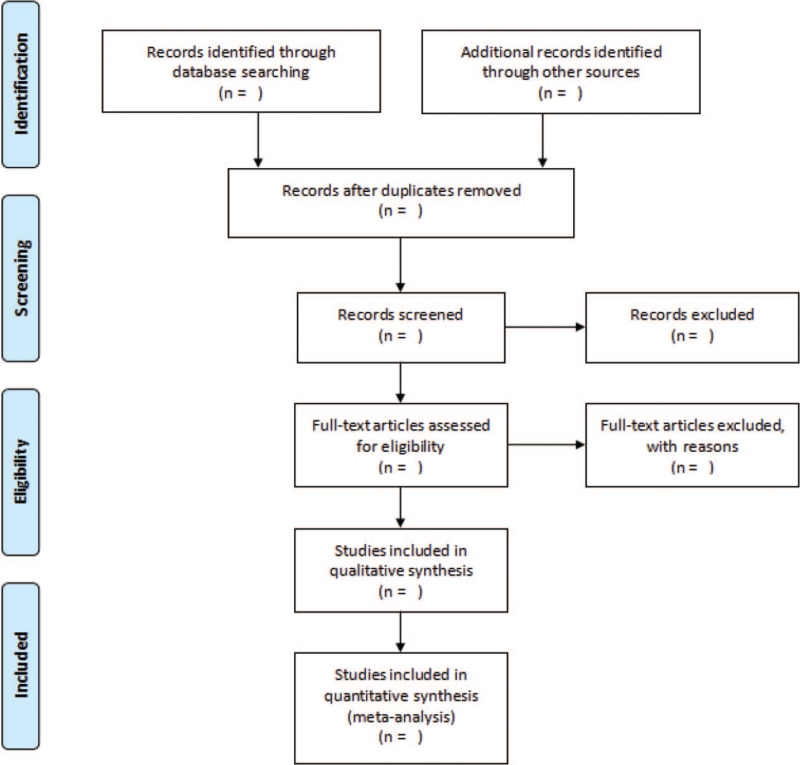
Trial flow chart.

The studies of electronic searches will be exported to EndNote V.9.1 software. Two authors will independently undertake the process of selecting the search results according to the inclusion and exclusion criteria. They will review and screen the titles and abstracts retrieved by literature search to exclude irrelevant trials. The causes of both selections will be documented and full texts will be obtained and checked for further evaluation if necessary. When there is uncertainty about the eligibility of the study, reviewers will arrive at a decision via discussion and consensus with a third reviewer. The selection process will be shown in a PRISMA flow diagram (Fig. 1).

### 3.5 Data extraction

Two researchers independently completed the evaluation of the research according to the conformity assessment form and extracted the research results. The extracted data includes author, gender, age, publication date, country, region, sample size, intervention details, follow-up information, safety, results, and so on. The above information will be repeatedly checked by 2 researchers, and any disputes about data extraction will be resolved through negotiation.

### 3.6 Risk of bias assessment

The Cochrane collaborative tools will be used to assess the risk of literature bias. Two investigators used RevMan 5.3.0 to assess method quality independently. Evaluate the following 7 aspects, including randomness, the blindness of participants and researchers, sequence generation, allocation hiding, blindness of result evaluation, selective result reporting, incomplete result data, and other biases. The quality of each experiment was assessed as low, unclear, or high biased. Resolve differences through discussion between the 2 reviewers or seeking third-party consultation.

### 3.7 Data synthesis

For the meta-analysis, RevMan Version 5.4 software (The Cochrane Collaboration, 2020) will be used to combine the relative risks for dichotomous outcomes and mean differences or standardized mean differences for continuous outcomes, with both having 95% confidence intervals. We will pool data across the studies for meta-analysis using random-effect or fixed-effect models.

### 3.8 Sensitivity analysis

Sensitivity analysis will be performed to determine the robustness of the review results with respect to the following aspects: impact of sample size, the effect of missing data, and methodological quality.

### 3.9 Subgroup analysis

If necessary, we will undertake a subgroup analysis based on the different patient demographics, study quality, types of interventions and comparators, and outcome measurements.

### 3.10 Assessment of heterogeneity

Statistical heterogeneity should be evaluated by Chi-Squared tests and *I*^2^ statistic. The results of the *I*^2^ statistic, which determine the use of the fixed-effects model or random-effects model, cover unimportant heterogeneity (0%–40%), moderate heterogeneity (30%–60%), substantial heterogeneity (50%–90%), and considerable heterogeneity (75%–100%). A random-effect model or subgroup analysis should be used when there exists significant heterogeneity.

### 3.11 Grading the quality of the evidence

Grading of Recommendations Assessment, Development, and Evaluation will be used to evaluate the quality of evidence for key outcomes. The quality of evidence will be graded into 1 of 4 levels: high, moderate, low, and very low.

### 3.12 Ethics and dissemination

Ethical approval is not necessary since this systematic review will be based on published research. The results of this review will be disseminated through peer-reviewed journal articles and conference presentations.

## 4 Discussions

Acupuncture combined with Chinese herbal medicine to treat DPN are easily accepted by the majority of patients due to avoiding the serious side effects of chemical drugs.^[16–18]^ Multiple RCTs have demonstrated the efficacy of acupuncture combined with Chinese herbal medicine in the treatment of DPN, however, the procedure of acupuncture combined with Chinese herbal medicine treatment on DPN has not yet been standardized and the quality of the clinical trials was uneven, as a result, clinicians tend to choose therapies based on their own clinical experience rather than the most effective treatment which is supported by evidence-based medicine. Network meta-analysis can evaluate and sequence a variety of different therapies directly or indirectly.^[19–21]^ To the best of our knowledge, this study will be the first network meta-analysis of acupuncture combined with Chinese herbal medicine for the treatment of DPN. The results of this study may be useful to clinicians, practice guide developers, researchers, decision-makers, and so on. Therefore, we hope that our results will provide credible evidence to support the clinical selection of acupuncture combined with Chinese herbal medicine and encourage wider acceptance of acupuncture combined with Chinese herbal medicine as a complementary and alternative medicine for DPN.^[22]^

## Author contributions

**Conceptualization:** Yong Shi.

**Data curation:** Le Liu.

**Funding acquisition:** Jundong Jiao.

**Software:** Xuefeng Sun.

**Supervision:** Jundong Jiao.

**Writing – original draft:** Yong Shi.

**Writing – review & editing:** Yong Shi.
